# Comparison of the clinical effects of white brand toothbrushes versus Vitis Suave®

**DOI:** 10.4317/jced.52490

**Published:** 2015-10-01

**Authors:** Maria Faus-Damiá, Marta Segarra-Vidal, Eduardo Lucas-Alcahuz, Andrés López-Roldán, Francisco Gil-Loscos, Francisco Alpiste-Illueca

**Affiliations:** 1 DDS, Master in Periodontics; 2 Associate Professor of Periodontics, University of Valencia; 3 Doctor in Odontology, Associate Professor of Periodontics, University of Valencia; 4 Professor, Supervisor of the subject of Periodontics, Director of the Master in Periodontics and Ossoeintegration, University of Valencia. Valencia, Spain

## Abstract

**Background:**

There has been an increase in the use of white label manual toothbrushes and a greater increase in inquiries for discomfort of the gingiva and mucosa.

**Material and Methods:**

A randomized, double-blind, cross-over clinical trial was made of four white brand toothbrushes versus a control brush (Vitis Suave®), with the recording of plaque index, bleeding upon probing, and gingival abrasions following utilization of the different brushes.

**Results:**

All the brushes except Deliplus® were equally effective in terms of plaque removal (*p*<0.05). Vitis Suave® and Veckia® were the brushes associated to the greatest increase in minor abrasions (*p*<0.01), while Veckia®, Carrefour® and Deluxe® significantly increased the number of medium intensity abrasions (*p*<0.05). These brushes also increased the number of large abrasions, though statistical significance was not reached in this case.

**Conclusions:**

The white brand brushes proved effective in controlling bacterial plaque, but were associated to more intense soft tissue abrasion.

** Key words:**Gingival abrasions, manual tooth brushing, white brand, clinical effects.

## Introduction

Bacterial plaque is the main cause of both caries and periodontal disease (1). Thorough and regular plaque removal therefore remains crucial for securing optimum oral health (2). In this regard, tooth brushing is the mechanical method most commonly used for eliminating dental plaque in the western world (3).

The literature describes a series of factors related to both plaque elimination capacity and to the risk of causing undesired effects secondary to brushing, such as brush design, individual brushing skill, the brushing technique used, and the frequency and duration of brushing. These last three factors are directly conditioned by patient motivation and the instructions received from the dental professional. Brush design in turn refers to the size and shape of the brush and handle, and the brush bristle specifications (4). In relation to bristle design, the material composition, number of bristles per unit surface area, and bristle thickness, length and morphology are very important, since they contribute to the hardness of the toothbrush.

**Figure 1:**
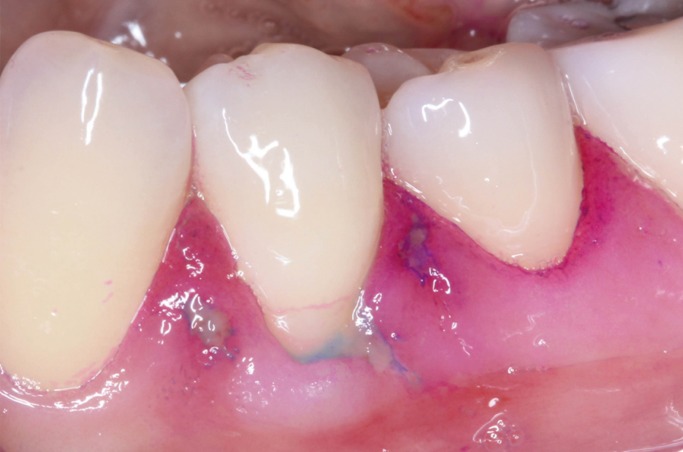
Clinical view of gingival abrasion and recession caused by traumatic brushing.

If the bristle characteristics are not suited to the specific conditions of the patient, brushing can result in dental (abrasion and/or sensitivity) and gingival damage (abrasions and/or recession) (5). In this regard, it is very common in clinical practice to observe recessions in patients with fine gingival biotypes, associated to traumatic brushing (Fig. 1).

Epithelial abrasion caused by brushing initially tends to be superficial, localized, reversible and often asymptomatic when affecting the gums, in contrast to abrasions that affect the epithelium of the alveolar mucosa. The underlying mechanism involves puncture or scratching trauma (shear forces) produced by the brush bristles on the superficial cells of the epithelium, and which can lead to partial or complete detachment of the epithelial surface (6). If trauma is more intense or repeated, or especially if the epithelial layer is thin (as in the case of the mucosa), abrasion can extend in depth to the submucosa, with exposure of the connective tissue (7). Such damage can be visualized by using stains such as toluidine blue or erythrosine (6,8,9). Over the long term, repeated trauma of this kind can give rise to more serious lesions, particularly at the gingival margin, such as inflammation of the gingival margin, cracks and recessions. Such lesions are very common in people who fail to use an adequately designed brush and apply too much pressure while brushing (10).

In 1931, Hirschfield found that when an adequate brushing technique is used, soft bristles with a rounded ending are less irritating than hard and sharp-tipped bristles. Posteriorly, Bass (1948) recommended round-tipped bristles to minimize the risk of gingival abrasions and even recessions (11). Breitenmoser *et al.* (1979) found that bevel-tipped bristles cause significantly greater gingival damage (30%) than rounded bristles (6). Alexander (1977) in turn observed that brushes with non-rounded bristle tips are twice as damaging for the soft tissues (12). Danser (1988) concluded that the extent of the gingival abrasions caused by brushing is more dependent upon a non-rounded shape of the bristle tips than on the force or pressure applied during brushing (5).

The working hypothesis of this study is based on our own clinical experience (Dental Clinic of the Faculty of Medicine and Dentistry, University of Valencia, Valencia, Spain). In effect, in recent years we have observed an increased use of manual white brand brushes among our patients. Some people have even replaced our initially recommended quality brushes with white brand toothbrushes. On the other hand, we observed an apparent relationship between increased consultation due to gingival and mucosal discomfort and the use of such white brand brushes.

Thus, the present study was carried out to explore the differences in bacterial plaque control and soft tissue abrasions using a manual toothbrush of established quality (Vitis Suave®, Dentaid) versus four white brand brushes sold in large shopping centers.

## Material and Methods

-Study sample

A prior study power analysis was carried out, assuming a basal probability of abrasions in the healthy population of 1%. An adequate sample size was taken to imply a difference of proportions power test result of over 0.8. The G*Power 3.1.3 statistical application was used for the analysis of power. Assuming a confidence level of 95%, a sample of 34 patients guaranteed a power of 0.9 in detecting a difference of proportions of 0.1 for the binomial test. A sample size between 34-44 patients guaranteed a statistical power of between 0.87-0.93 for detecting as significant an effect size of 0.5 using the Wilcoxon test.

The study was carried out in abidance with the principles of the Declaration of Helsinki (1975) , and was approved by the Clinical Research Ethics Committee of the University of Valencia. The study subjects were volunteers (fourth-year dentistry students of the Faculty of Medicine and Dentistry, University of Valencia), and all gave written informed consent to participation in the study.

The participants were all over 18 years of age, periodontally healthy (with no gingivitis of clinical attachment loss), and had at least 6 teeth per quadrant. Pregnant or nursing women were excluded, as were smokers, individuals using medications or with systemic diseases known to have an impact upon periodontal health, and subjects with dental implants, removable or fixed dentures, or retainers or orthodontic brackets. Likewise, individuals using oral rinses or antiseptics in the 15 days before or during the study were excluded, as were people using antibiotics in the month before or during the study, people with a fine gingival biotype, soft tissue sensitivity or recessions affecting any of the teeth, acute intraoral mucosal lesions or a history of recurrent aphthae, and those with manual brushing skill difficulties.

-Study design

A randomized, double-blind, crossover clinical trial was made involving two calibrated explorers blinded to the type of brush used. We evaluated a control brush (Vitis Suave®, Dentaid) and four white brand brushes:

• Veckia®: sold by the department store chain El Corte Ingles. Hardness was not specified by the manufacturer.

• Deliplus®: sold by the supermarket chain Mercadona, with three hardness levels (hard, medium and soft). The soft brush was used in this study.

• Carrefour®: sold by the supermarket chain Carrefour. Hardness was not specified by the manufacturer.

• Deluxe®: sold by Chinese bazaar shops. Hardness was not specified by the manufacturer.

The ultrasoft toothbrush Vitis Ultrasuave® (Dentaid) was employed as initial brush to minimize possible lesions. At the start of the study, the participants were instructed to use the modified Bass brushing technique during two minutes. Each study brush was used three times a day for three days, and with a 15-day washout period between each study brush. During these washout periods Vitis Ultrasuave® (Dentaid) was used to ensure disappearance of the possible lesions caused.

In order to prevent the sequence of brushes used from influencing the results of the study, the sequence was randomized using a Microsoft Excel table. A total of 120 possible brush sequences were generated, with random assignment to each participant carried out by a person unrelated to the study. The brushes were coded as follows: A. Vitis Suave, B. Veckia, C. Deliplus, D. Carrefour, E. Deluxe. At the end of the study, all the subjects had used all the brushes.

A full periodontal examination was made at the following timepoints:

• Before the start of the study (pre-study clinical examination).

• After initial prophylaxis (rubber cup and ultrasound tartar removal) and Vitis Ultrasuave® use for 15 days to minimize the lesions caused by prophylaxis (basal clinical examination). Professional prophylaxis was then again performed (rubber cup and prophylactic toothpaste), and the patients received instructions on oral hygiene and were randomized to one of the 5 study brushes.

• After each of the study brush utilization periods. Professional prophylaxis was then again performed (rubber cup and prophylactic toothpaste), and the patients received instructions on oral hygiene and again made use of the Vitis Ultrasuave® brush for 15 days in order to minimize the lesions caused by the study brush.

• After each washout period. Professional prophylaxis was then again performed (rubber cup and prophylactic toothpaste), and the patients received instructions on oral hygiene and were randomized to one of the 5 study brushes.

**Figure 2:**
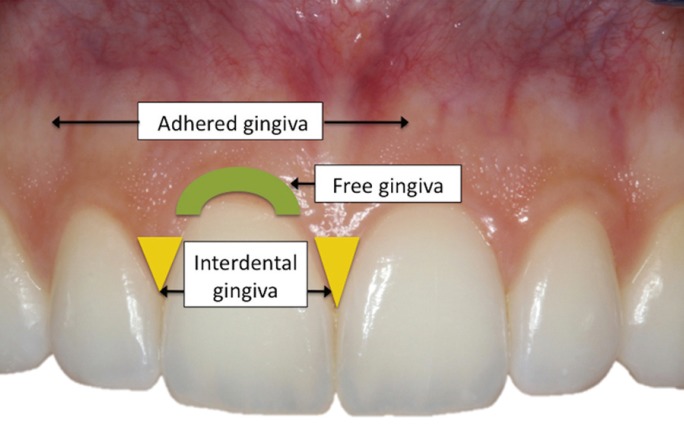
Distribution of the gum areas in relation to the location of gingival abrasion.

The following parameters were evaluated:

• Silness and Löe gingival index

• Gingival recession

• Bleeding upon probing (BOP)

• Quigley and Hein plaque index

• Gingival abrasions. The diameter of abrasion along its long axis was measured using a periodontal probe (Williams PQ-OW 208 396, Hu-Friedy®; Rotterdam, The Netherlands) and following the classification of Danser: 5 minor abrasion: diameter ≤ 2 mm; medium: diameter ≥ 3 mm and ≤ 5 mm; and large: diameter >5 mm. We also considered the location of the abrasion within each arch (anterior, premolar, molar) and with respect to the affected area (gingival: free and interdental gum; cervical: adhered gum) (Fig. 2).

The clinical measurements were made by two calibrated explorers (agreement 97%, margin of error 1 mm and 95% confidence interval). A descriptive statistical study was made of the plaque index (mean plaque index at 6 points of each tooth in each zone) and the number of minor, medium and large abrasions, per zone and location.

The bivariate comparative study in turn was based on the Kolmogorov-Smirnov normality test, the student t-test for independent samples, repeated measures analysis of variance (ANOVA) F-test, and the Wilcoxon test for two independent samples. The Friedman, McNemar and Kruskal-Wallis tests were used for the comparison of more than two independent samples. Statistical significance was considered for *p* < 0.05.

## Results

A total of 5 brushes were evaluated in 38 patients. The brushes were assigned on a randomized basis, and the results obtained were compared with the data collected on occasion of the basal clinical examination.

-Effect of Vitis Ultrasuave® as washout brush

A first evaluation the effect of the Vitis Ultrasuave® as washout brush revealed a significant difference in terms of plaque removal at premolar level (1.33 ± 0.63 vs 1.08 ± 0.44 at the initial and basal examination, respectively). A decrease was observed in the anterior and molar zones, though statistical significance was not reached (anterior zone 1.30± 0.75 vs 1.12± 0.48; molar zone 1.8 ±0.71 vs 1.61±0.55).

No significant change in terms of abrasion was recorded (anterior zone 0.39 vs 0.40 at the initial and basal examination, respectively; premolar zone 0.53 vs 0.71, respectively; molar zone 0.53 vs 0.76, respectively).

-Comparative analysis of the study brushes

• Plaque index

In the anterior zone all the brushes except Deliplus® (1.27) produced significant or very notorious reductions in dental plaque (Veckia® 1.15, Deluxe® 1.10; *p* < 0.05; Vitis Suave® 1.16, Carrefour® 1.17; 0.05 < *p* < 0.1).

In the premolar zone, Vitis Suave® was found to be the most effective brush in reducing the plaque index (1.08; *p* < 0.01). With the exception of Deliplus® (1.24; *p* > 0.05), the rest of the brushes also yielded positive results (Veckia® 1.16, Deluxe® 1.13; *p* < 0.05; Carrefour® 1.15; 0.05 < *p* < 0.1).

In the molar zone, only Vitis Suave® and Carrefour® produced significant plaque reductions (1.57 and 1.30, respectively; *p* < 0.05).

Without distinguishing tooth position, all the brushes except Deliplus® were found to be equally effective in terms of plaque removal (Vitis Suave® 1.50 ± 0.64, Veckia® 1.30 ± 0.51, Carrefour® 1.30 ± 0.44, Deluxe® 1.26 ± 0.44; *p* < 0.05; Deliplus® 1.41 ± 0.55) ( Table 1).

**Table 1:**
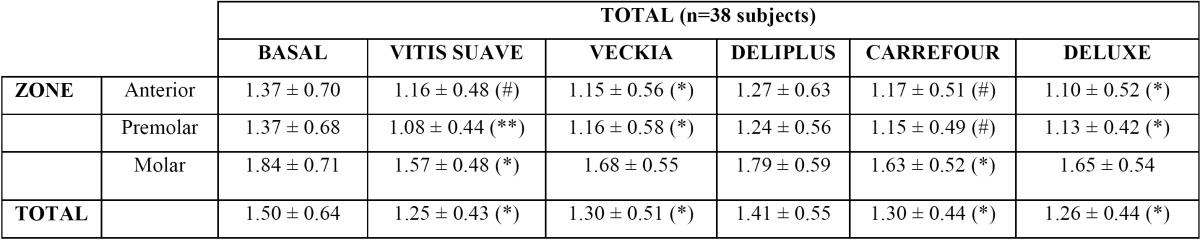
Plaque index divided by oral zones after use of the different study brushes.

• Gingival abrasions

The differences in gingival abrasion with each of the study brushes were evaluated with respect to the basal recordings. In the zone anterior, Vitis Suave® and Veckia® caused the greatest increase in minor abrasions (0.66 and 0.24, respectively vs 0.18; *p* < 0.05), while Deluxe® was associated to the greatest increase in medium abrasions – though statistical significance was not reached (0.26; 0.05 < *p* < 0.1). In turn, large abrasions were more frequent with Carrefour®, though here again significance was not reached (0.03; *p* > 0.05).

In the premolar zone, Vitis Suave® and Veckia® again caused the greatest increase in minor abrasions (0.76 and 0.68, respectively; *p* < 0.01). All the brushes significantly increased the number of medium intensity abrasions (Veckia® 0.32, Deliplus® 0.26, Carrefour® 0.26, and Deluxe® 0.21; *p* < 0.05), with the exception of Vitis Suave®, which caused fewer medium abrasions (0.08; 0.05 < *p* < 0.01). All the brushes produced large abrasions, except Deliplus®, though statistical significance was not reached (Vitis Suave® 0.03, Veckia® 0.08, Carrefour® 0.03, Deluxe® 0.13; *p* > 0.05). No statistically significant differences were recorded in the molar zone.

**Figure 3:**
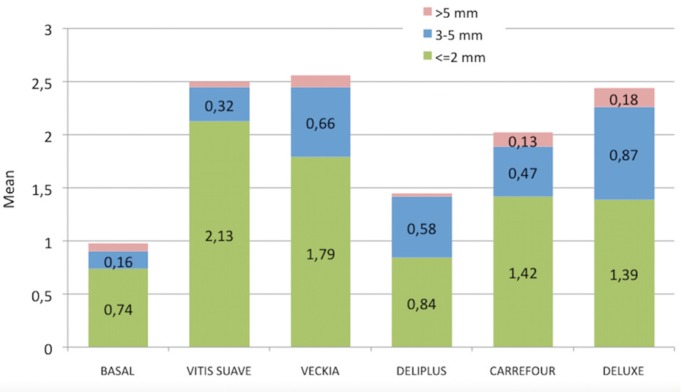
Distribution of gingival abrasion in relation to the type of brush used.

Globally and independently of the zone, Vitis Suave® and Veckia® were the brushes that produced the greatest increase in minor abrasions (2.13 and 1.79 respectively; *p* < 0.01). Veckia®, Carrefour® and Deluxe® significantly increased the medium intensity abrasions (0.66, 0.47 and 0.87, respectively; *p* < 0.05) and also increased the presence of large abrasions – though in this case significance was not reached (0.11, 0.13 and 0.18, respectively; *p* > 0.05) (Fig. 3).

## Discussion

The present study was designed to evaluate the possible differences in plaque control and gingival damage between a manual toothbrush of established quality (Vitis Suave®) and four white brand brushes. The results found the latter to produce a greater increase in medium and large abrasions, which are the lesions that can have a negative impact upon gingival health. The observation that Vitis Suave® (taken to be the reference brush) and Veckia® were the toothbrushes that caused the largest increase in minor abrasions is of questionable relevance, since the distinction of minor abrasions is no simple task even for trained and cali-brated explorers. Furthermore, according to the clinical observations made, minor abrasions do not imply a serious problem for the gums.

Few studies have evaluated white brand brushes. Van Nüss *et al.* (2010) found few differences in bristle wear among brushes of different prices, but did not analyze their effectiveness in terms of plaque control or possible gingival damage (13).

Pimentel *et al.* (2003) analyzed the effectiveness of low-cost brushes with and without toothpaste in removing bacterial plaque, though in patients with primary dentition. They observed no significant differences between the brushes in terms of the reduction of bacterial plaque.

One of the limitations of the present study is the fact that the participants were dental students. The findings therefore cannot be extrapolated to the general population. We decided to use this type of sample in order to minimize the human factor and ensure that the differences in the effects of the brushes were attributable to changes in toothbrush type – thereby controlling the bias inherent to the use of a heterogeneous sample of subjects.

After confirming that the 5 brushes produced different clinical effects, we considered the possibility that the order in which the different brushes are used may result in different effects (14,15). As a first step, we had to ensure that all the brushes were randomized on a balanced basis over the 5 periods of the sequence. The chi-squared test was used to this effect, ensuring that the proportion of brushes A, B, C, D, E was homogeneous at all times. We were thus able to study the effect of brushing order, comparing the results with guarantees that the results obtained for a given brush type were not attributable to chance.

**Figure 4:**
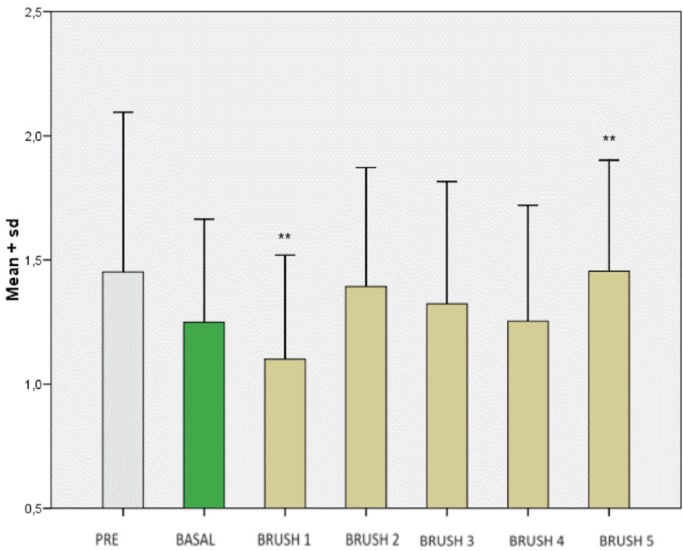
Mean plaque index on the study visits.

There were no significant differences in plaque index after using brushes A, B, C, D and E (*p* = 0.093), though a more or less continuous tendency towards increased plaque index values was noted from the start to the end of the study (Fig. 4).

**Figure 5:**
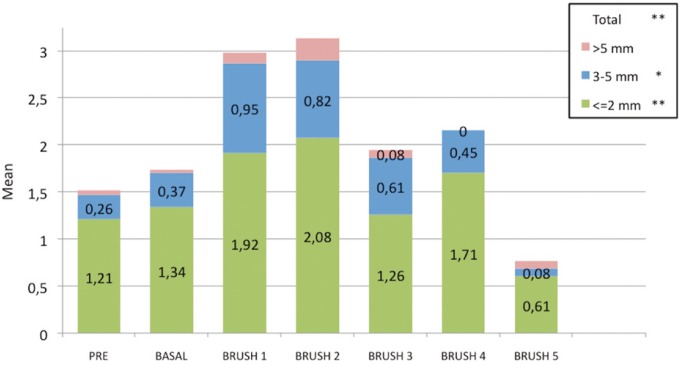
Distribution of the gingival abrasion on the study visits.

Regarding the number of abrasions, we observed a significant decrease in the number of minor, medium and total abrasions as the sequential use of the different brushes progressed (Fig. 5). The explanation for this could be patient tiredness or loss of motivation, since at the end of the study the participants had completed a total of 11 visits. This hypothesis is also supported by the fact that the number of call to patients who failed to report to the visits increased by 17% in the course of the study.

As dental professionals, we must assess the quality of a brush on the basis of its capacity to eliminate bacterial plaque without causing adverse effects upon oral tissue health and integrity. This in turn is closely related to bristle morphology. In this regard, in 1988 Silverstone and Featherstone classified manual toothbrushes as either acceptable or not acceptable, depending on the percentage of round-tipped bristles they contain (16).

The current economic crisis is probably the reason for aggressive competition among manufacturers, with the introduction of new white brand brushes that are cheaper and more accessible to the general population (Pimentel *et al.*, 2003)(17). However, the design of these brushes is not warranted or backed by studies of any kind - the manufacturer sales strategy being simply to offer a cheaper product.

Despite the limitations of our study, the following conclusions can be drawn:

1. With the exception of Deliplus®, the rest of the brushes were found to be effective in controlling bacterial plaque.

2. The white brand brushes – particularly Deluxe® – were associated to larger abrasions. Most of the abrasions were located in the free and interdental gums.

3. Vitis Ultrasuave® afforded important plaque control and moreover produced no gingival abrasions.

4. The order in which the brushes were used in the course of the study led to certain differences in the clinical results obtained. In this regard, the number of abrasions gradually decreased as the sequential use of the different brushes progressed. Likewise, a greater presence of plaque was noted in the later stages of the study. This appears to reflect a loss of patient motivation over time.

Although further studies are needed before these conclusions can be applied in clinical practice, our results suggest that a soft or ultrasoft brush (Vitis, Dentaid) is best for patient gingival health, since it allows adequate removal of bacterial plaque without producing important gingival lesions. In this respect, the recommendation of an ultrasoft toothbrush may be of great clinical interest.
